# Phytoextraction of Heavy Metals: A Promising Tool for Clean-Up of Polluted Environment?

**DOI:** 10.3389/fpls.2018.01476

**Published:** 2018-10-16

**Authors:** Jachym Suman, Ondrej Uhlik, Jitka Viktorova, Tomas Macek

**Affiliations:** Department of Biochemistry and Microbiology, Faculty of Food and Biochemical Technology, University of Chemistry and Technology, Prague, Czechia

**Keywords:** heavy metals, phytoremediation, phytoextraction, hyperaccumulators, genetically modified plants, heavy metal protein transporters, heavy metal binding proteins, green biotechnology

## Abstract

Pollution by heavy metals (HM) represents a serious threat for both the environment and human health. Due to their elemental character, HM cannot be chemically degraded, and their detoxification in the environment mostly resides either in stabilization *in situ* or in their removal from the matrix, e.g., soil. For this purpose, phytoremediation, i.e., the application of plants for the restoration of a polluted environment, has been proposed as a promising green alternative to traditional physical and chemical methods. Among the phytoremediation techniques, phytoextraction refers to the removal of HM from the matrix through their uptake by a plant. It possesses considerable advantages over traditional techniques, especially due to its cost effectiveness, potential treatment of multiple HM simultaneously, no need for the excavation of contaminated soil, good acceptance by the public, the possibility of follow-up processing of the biomass produced, etc. In this review, we focused on three basic HM phytoextraction strategies that differ in the type of plant species being employed: natural hyperaccumulators, fast-growing plant species with high-biomass production and, potentially, plants genetically engineered toward a phenotype that favors efficient HM uptake and boosted HM tolerance. Considerable knowledge on the applicability of plants for HM phytoextraction has been gathered to date from both lab-scale studies performed under controlled model conditions and field trials using real environmental conditions. Based on this knowledge, many specific applications of plants for the remediation of HM-polluted soils have been proposed. Such studies often also include suggestions for the further processing of HM-contaminated biomass, therefore providing an added economical value. Based on the examples presented here, we recommend that intensive research be performed on the selection of appropriate plant taxa for various sets of conditions, environmental risk assessment, the fate of HM-enriched biomass, economical aspects of the process, etc.

## Introduction

With the industrialization and urbanization of developing countries and the increasing demands of mankind, the consumption of heavy metal elements (HM, see Glossary) has been growing enormously. As a result, pollution by HM and metalloids (see Glossary) has become an increasingly serious issue, which has raised public awareness. Unlike organic pollutants, which can be entirely, or partly degraded by the action of various (micro)organisms or detoxified by the action of plants, HM are non-degradable and can only be transformed into forms with altered toxicity and/or mobility/bioavailability (e.g., bacterial reduction of Hg^2+^ to elementary Hg^0^ that is less toxic and can evaporate, or the reduction of arsenate to less toxic arsenite oxyanions; Silver and Phung, 2005; Silver and Phung le, 2005). The persistence of HM in environmental matrices, especially soils, sediments and water bodies, is a result of the elemental character of HM (Chapman et al., 1996). Pollution by HM therefore represents a long-term threat for the environment and human health, not only in industrial and post-industrial areas.

**Glossary**

**Heavy Metal** There is not yet a consensus definition of the term “heavy metal” (HM). Nevertheless, the most recent criterion suggested defines HMs as naturally occurring metals having atomic number >20 and elemental density >5 g.cm^−3^ (Ali and Khan, 2018). In biological sciences, this term instead describes a series of metallic elements and metalloids with a potential toxic effect on living organisms even at very low concentrations. From this toxicological/ecotoxicological standpoint, chemical properties and behavior in living systems are a more important criterion. Therefore, in addition to transition metal elements such as Cd, Pb, Hg, Zn, Cu, Co, Ni, Al, Cr, Fe, the metalloids As, Se or Sb can also be regarded as “heavy metals.”

**Phytoremediation** The use of plants for the restoration of a polluted environment. Generally, there are two principal strategies for decreasing HM toxicity in soils: *site stabilization* (including phytostabilization techniques) and “*clean-up” techniques* (including phytoextraction and phytovolatilization techniques).

**Phytostabilization** The use of plants for the reduction of HM toxicity in soil through reducing their mobility/bioavailability.

**Phytoextraction** The use of plants for HM removal from the contaminated matrix (soil and water) through their uptake into the harvestable parts of the plant.

**Hyperaccumulation** The ability of a plant to accumulate HMs in its above-ground parts without phytotoxic symptoms at concentrations up to 100–1,000-fold higher than in non-hyperaccumulating species (Baker, 1981). Such an ability resides in (i) enhancement of the uptake of HMs from the rhizosphere across the root cell plasma membrane, (ii) reduction of the sequestration of HMs in root-cell vacuoles, (iii) enhancement of the loading of HMs into the xylem for transport to shoots, and (iv) stimulation of HM influx across the leaf cell plasma membrane and sequestration in the leaf (mesophyll cells) vacuoles (Milner and Kochian, 2008).

**Phytomining** The use of plants for the *in situ* removal of HMs from sub-economic ore bodies, metalliferous soils or from contaminated mine sites with the additional aim of recovering an economic amount of metals from the plants [according to Sheoran et al. (2009)].

**“Gene-Stacking” Strategy (also “Gene-Pyramiding” Strategy)** The expression or manipulation of multiple (trans)genes in a single plant line [for review see Halpin (2005)]. This process provides the potential to accumulate multiple traits, typically multiple resistance to different types of stress or pathogen. As discussed in the text, this can also provide a tool for obtaining a plant variety suitable for efficient HM phytoextraction.

**Short-Rotation Forestry/Plantation System** The concept of culturing woody plants (especially fast-growing trees such as willow, poplar, pine, aspen, birch, beech, eucalyptus, etc.) or annually-harvested energy crops for energy production.

**Trangenosis** A gene-technology breeding method of introducing a *transgene*, which is according to Jacobsen and Schouten (2009) a (synthetic) gene with some or all regulatory sequences and coding sequences from donors other than crossable plants, including microorganisms and animals. Transgenes comprise a new gene pool for plant breeding.

**Cisgenesis** A gene technology breeding method of introducing a *cisgene*, which is according to Jacobsen and Schouten (2009) an already existing natural gene from the plant taxon itself or from crossable species, including its native promoter and terminator. The gene belongs to the traditional breeder gene pool and is the already existing result of natural evolution.

**Intragenesis** A gene technology breeding method of introducing an *intragene*, which is according to Jacobsen and Schouten (2009) a gene comprising of natural functional elements, such as the coding part, promoter and terminator originating from different genes from the plant taxon itself or from crossable species. All natural gene elements belong to the traditional breeder gene pool.

Conventional physical and chemical methods of HM removal from a polluted environment are usually not usable at large scales, and are often costly and not well accepted by the public [for a recent review see Khalid et al. (2017)]. In contrast, bioremediation i.e., the use of living organisms or their parts for remediation, is a group of methods that are highly applicable in large contaminated areas, especially in cases where the removal of HM contamination is not urgent. The use of plants for the decontamination of a polluted environment has been termed phytoremediation and was described in the mid-90s (Sandermann, 1994; Cunningham et al., 1995; Schnoor et al., 1995; Cunningham and Ow, 1996). Ever since, a vast number of studies have focused on the employment of various plant species for the removal or stabilization of both organic and inorganic pollutants (for reviews see Salt et al., 1998; Macek et al., 2000; Mackova et al., 2006a,b; Marmiroli et al., 2006; Cristaldi et al., 2017; Wang et al., 2017).

Generally, the most yet-employed strategies on how to remediate an HM-polluted environment are based either on their stabilization *in situ*, i.e., reducing HM acute toxicity through decreasing their mobility and bioavailability (so-called “site stabilization” techniques), or are based on HM removal (the group of “clean-up” techniques). When using plants, the first group is referred to by the term *phytostabilization* (see Glossary). Among the clean-up techniques, *phytoextraction* employs plants for the extraction of HM from soils and their accumulation in harvestable parts, which are subsequently removed and further processed (Baker et al., 1994; Salt et al., 1995, 1998; Chaney et al., 1997). *Phytovolatilization*, another subgroup of clean-up techniques, represents the conversion of HM/metalloids such as Hg, As, and Se into a volatile form via biological conversion within plants, and their release into the atmosphere (Rugh et al., 1996; Raskin et al., 1997; He et al., 2001; Che et al., 2003; Tangahu et al., 2011). Despite decreasing the pollution locally, this group of techniques contributes to the pool of mobile forms transportable over long distances, and is somewhat controversial (Prasad and Freitas, 2003).

Over the last three decades, great progress has been made in researching the applicability of phytoremediation techniques, including the use both of natural varieties and genetically modified (GM) plants. Therefore, in this review, we discuss the use of plants for the phytoextraction of HM and metalloids from soils. Particular attention is paid to the prospective employment of genetic modifications leading to plant varieties with a phenotype favoring HM phytoextraction from polluted sites.

## Hyperaccumulators

Baker (1981) defined two main strategies adopted by plants naturally growing on metalliferous sites based on the strategy of survival. *Excluders*, representing the majority of plant species capable of surviving in soils containing elevated levels of toxic trace elements, have adopted the survival strategy of maximal exclusion of HM ions from the plant. If an HM ion is taken up by an excluder plant, the toxic effect is restricted to the roots, where it is detoxified, while the aerial parts remain more or less unaffected. In contrast, *hyperaccumulators* are plants which, when exposed to elevated concentrations of HM, are able to accumulate them in their above-ground parts without phytotoxicity symptoms (Baker, 1981; Rascio and Navari-Izzo, 2011; van der Ent et al., 2013). The term *hyperaccumulator* was first applied when describing the New Caledonian Ni-accumulating tree *Sebartia acuminata* (*Sapotaceae*); the nickel content in the latex was determined to be 26% percent of dry weight (Jaffré et al., 1976). According to Baker and Brooks (1989), the content limits of some metal elements in dry biomass for plants to be termed hyperaccumulators are 100 mg.kg^−1^ for Cd and Se, 1,000 mg.kg^−1^ for Co, Cu, Ni and Pb, and 10,000 mg.kg^−1^ for Zn and Mn. These values are up to 100–1,000-fold higher than for non-hyperaccumulating species under the same conditions (Reeves, 2006; Rascio and Navari-Izzo, 2011). The number of identified hyperaccumulators has been constantly rising, with over 450 HM-hyperaccumulating species known as of 2015, found in 45 angiosperm families (Rascio and Navari-Izzo, 2011; Bhargava et al., 2012; Krzciuk and Gałuszka, 2015). About 25% of hyperaccumulators identified so far recruit from the family *Brassicaceae*; other families rich in hyperaccumulators include *Asteraceae, Euphorbiaceae, Rubiaceae, Fabaceae, Scrophulariacea, Myrtaceae, Proteaceae, Caryophylaceae, Tiliaceae*, etc. (Reeves, 2006; Rascio and Navari-Izzo, 2011). Some plant genera appear to include a significant proportion of hyperaccumulator species; e.g., in studies comprehensively surveying the genus *Alyssum*, 48 out of 170 species tested were found to hyperaccumulate Ni (Brooks and Radford, 1978; Brooks et al., 1979). Interestingly, hyperaccumulation ability was found to vary within a species; metal specificity and accumulation can vary among different populations (Reeves et al., 2001; Assuncao et al., 2008).

As stated by Milner and Kochian (2008), who surveyed the Zn, Cd and Ni model hyperaccumulator alpine pennycress (*Noccaea caerulescens*, formerly *Thlaspi caerulescens*), several crucial physiological steps of HM detoxification are different in hyperaccumulators compared to non-hyperaccumulators. These are (i) boosted HM ions uptake from the rhizosphere across the root cell plasma membrane, (ii) reduced HM ions sequestration in root vacuoles, (iii) intensified HM ions loading into the xylem for transport to shoots, and (iv) stimulated HM ions influx across the leaf cell plasma membrane and (v) sequestration in the leaf vacuole. Nevertheless, a considerable contribution of cell wall components, especially low-methylesterified pectins, in the sequestration of HM ions within a plant body was also proposed (for reviews, see Krzesłowska, 2011; Le Gall et al., 2015). Leitenmaier and Küpper (2013) concluded that the key factor for hyperaccumulation is the plant's enhanced active metal transport rather than strengthened metal complexation by various intracellular ligands (such as glutathione, phytochelatins, or metallothioneins). Similarly, cross-species transcriptomic and genome-wide analyses of the extremophilic Zn- and Cd-hyperaccumulator *Arabidopsis halleri* revealed an enrichment in HM-transporting P-ATPAse *HMA4* gene copies and corresponding transcripts as well as other transition metal homeostasis and biotic stress function genes compared to the non-accumulating sister species *Arabidopsis lyrata* and closely related reference model species *Arabidopsis thaliana* (Hanikenne et al., 2008; Suryawanshi et al., 2016). More detailed information on the various tolerance strategies of plants under HM stress and on hyperaccumulators, their taxonomic and geographical distribution, metal specificity as well as molecular mechanisms behind hyperaccumulation have been provided by Reeves (2006); Verbruggen et al. (2009); Rascio and Navari-Izzo (2011); Leitenmaier and Küpper (2013); van der Ent et al. (2013); Krzciuk and Gałuszka (2015), and Singh et al. (2016).

## Phytoextraction–a tool for the remediation of HM-polluted sites?

In order to be suitable for phytoextraction purposes, plant species should meet the following criteria: (i) metallotolerance toward elements present in toxic levels, (ii) high biomass production and (iii) effective accumulation of HM in easy-to-harvest parts (Vangronsveld et al., 2009). The overall concept of the restoration of polluted areas using phytoextraction consists of the cultivation of appropriate plant species *in situ*, harvesting the HM-containing biomass and treating it to decrease its volume and weight (by composting, compacting, drying, thermal decomposition). The resultant HM-enriched mass containing high levels of metal contaminants is subsequently disposed of as a hazardous waste or, if economically advantageous, can be utilized for the re-extraction of trace elements (McGrath et al., 2002; Sheoran et al., 2009). In general, three basic strategies can be considered for HM phytoextraction, differing in the type of plant species used: (i) natural hyperaccumulators, (ii) fast-growing plant species with high-biomass production, and (iii) genetically engineered plants, all of which are discussed below. Advantages and drawbacks of these strategies are summarized in Table 1.

**Table 1:** Benefits and drawbacks of HM phytoextraction employing natural hyperaccumulators, high-biomass producing non-hyperaccumulators or genetically engineered plants.

	**Natural hyperaccumulators**	**High-biomass producing non-hyperaccumulators**	**Genetically engineered plant**
Advantages	High bioaccumulation rates Often autochtonic species–prevents the introduction of non-native and potentially invasive species	High biomass production rate, possibility of production of biomass with added value Low growth requirements Possible use in short-rotation plantation Number of species convenient for diverse range of climatic conditions, water regime, soil type, character of contamination Often autochthonic species–prevents the introduction of non-native and potentially invasive species Usually low metal/toxicant specificity–applicability for mixed contamination (both multiple HM and mixture of HM and organic xenobiotics)	Possibility of directed engineering of metallophenotype toward high bioconcentration factor Number of (trans)genes available for modification, possibility of organ/tissue-specific transgene expression enabling modulation of resultant phenotype Possible stacking of multiple phenotypical traits Possibility of selection of host plant species (depending on the intended fate or use of resulting HM-enriched biomass)
Disadvantages	High metal specificity (often only single heavy metal element hyperaccumulated) Often slowly growing, low-biomass producing species, with specific ecology and requirements in terms of climate, soil characteristics, water regime, etc.	Low bioaccumulation rates–lengthy phytoextraction process	Often high metal specificity depending on (trans)gene/genetic modification Lack of information from field trials performed on large areas in terms of overall applicability (economical aspects, efficiency of the process, management of HM-enriched biomass produced) GMO-linked environmental risks and related strict GMO regulation policy, thorough ecological risk assessment needed

Several criteria should be taken into account when evaluating the suitability of a plant species for HM phytoextraction, in particular the quantitative ability of each plant species to accumulate HM in harvestable parts, and the overall production of biomass which can be expressed by the biomass yield per crop and number of crops per year. McGrath and Zhao (2003) determined that for economically feasible phytoextraction, the use of a plant with a metal bioconcentration factor, defined as the ratio of HM concentration in the plant to the soil, higher than 20 and biomass yield of 10 t·ha^−1^ is required, or with a bioconcentration factor of 10 and biomass production of 20 t·ha^−1^ (Peuke and Rennenberg, 2005). Other criteria to be considered are the depth of the contaminated layer (if the contamination is deeper than 20–30 cm, deep-rooting woody species should be utilized), the intended fate of HM-enriched biomass (e.g., re-extraction of metal elements; biofuel, energy and fiber production), climatic and soil conditions, etc. (Brooks et al., 1998; Mench et al., 2009; Sheoran et al., 2009).

The toxic element and its bioavailable concentration in the polluted matrix (soil) also needs to be considered, as well as the ability of a plant to maintain photosynthetic activity and biomass production even under HM-induced stress. If a toxicant content is too high, it can exceed the plant's tolerance level, especially for non-hyperaccumulator species, resulting in toxic symptoms and reduced photosynthetic capability and, consequently, lower biomass yield and reduced phytoextraction performance of a plant. Therefore, a systematic experimental assessment of HM-induced alterations in biomass production, plant anatomy, morphology, photosynthetic, and transpiration rate, photosynthetic pigments content, GSH-content, activity of reactive oxygen species (ROS)-defense enzymes, etc. should be preferably performed during the evaluation of a plant's suitability for HM phytoextraction, as was done with poplar (*Populus* sp.) (Ivanova et al., 2011; Chandra and Kang, 2016), eucalyptus (*Eucalyptus camaldulenses*) (Gomes et al., 2012), flax (*Linnum ussitatissimum*) and China aster (*Callistephus chinensis*) (Kavuličová et al., 2012), cardoon (*Cynara cardunculus*) (Arena et al., 2017; Sorrentino et al., 2018), rapeseed (*Brassica napus*) (Benáková et al., 2017), etc.

### Phytoextraction employing high-biomass non-accumulators

The employment of fast-growing deep-rooting woody species such as willow (*Salix* spp.) and *Populus* spp. is a reasonable choice for phytoextraction (Unterbrunner et al., 2007). These taxa are able to produce a high amount of biomass in a relatively short time, have a high rate of water uptake and transpiration, have a deep root system, can be effectively and rapidly reproduced through re-sprouting, and their aboveground parts are easy to harvest. Importantly, Unterbrunner et al. (2007) discovered that Zn and Cd are preferentially accumulated in the leaves and not in the wood of these taxa; HM can be therefore continuously removed from the site by harvesting leaf biomass. Additionally, covering the remediated area with hardy vegetation prevents the erosion and transfer of contaminated material to the surrounding environment (i.e., plants act as phytostabilizers). Another major advantage of using non-edible woody species for HM clean-up is the biomass produced during the phytoextraction process which can provide an added economic value if used, for instance, in energy production (*short-rotation forestry system concept*; Einspahr, 1976a,b; Nixon et al., 2001; Volk et al., 2004; Hinchee et al., 2009; Kidd et al., 2015). For instance, experiments with cotton (*Gossypium* sp.) plants grown in a former industrial area in Bulgaria near Plovdiv contaminated with HMs demonstrated that after treating the produced cotton fiber with boiling water, the Cd, Zn, Cu, and Pb content was diminished to levels comparable to the plant material grown in non-contaminated sites. These findings make cotton another crop suitable for sustainable long-term use in contaminated areas (Yankov et al., 2000). Vangronsveld et al. (2009) demonstrated the potential of biomass for biofuel and biogas production despite the fact that the phytoextraction efficiency in their field trial, in which they employed *Z. mays*, tobacco (*Nicotiana tabacum*), *B. napus* and sunflower (*Helianthus annuus*), was not very encouraging (the estimated time required for clean-up of agricultural land moderately contaminated with Cd and Zn ranged from 29 years for tobacco to 234 years for rapeseed). An interesting approach to the subsequent utilization of HM-contaminated biomass produced during a phytoextraction process was applied in the studies of Zheljazkov and Nielsen (1996a,b). Medicinal plants such as peppermint (*Mentha piperita*) and lavender (*Lavandula angustifolia*) were proven to accumulate moderate levels of HM in the harvested biomass, and at the same time the aromatic oil produced from this contaminated biomass was contaminant free.

Several different clones of willows have been found to accumulate substantial amounts of Cd and Zn (Greger and Landberg, 1999). A field experiment performed with a Cd-accumulating clone of *Salix viminalis* cultivated in sludge-amended soil demonstrated that the total amount of extracted Cd (expressed as weight of Cd removed per ha and y) was ca 5 × and 6 × higher than the hyperaccumulating species *Alyssum murale* and *N. caerulescens*, respectively (Greger and Landberg, 1999). A large-scale field experiment performed on a site moderately contaminated with Cd and Zn in Lommel (Belgium) employed different clones of *Populus* sp. and *Salix* sp. for the phytoextraction of Cd. According to the most optimistic estimation based on the output data, from 12.5 up to 25 years would be needed for the restoration of a site with a Cd content of 1 mg·kg^−1^ soil (experiments reviewed in Vangronsveld et al., 2009). The influence of clone choice and the necessity of foliage harvesting were also highlighted in the two above-mentioned studies, as well as in the study of Vyslouzilova et al. (2006). Another field trial employing a short-rotation coppice of willow grown in metal-contaminated agricultural soil demonstrated the ability of a *Salix* coppice to remove 72 g Cd and 2 kg Zn ha^−1^ year^−1^, values much higher than maize (*Zea mays*) or *B. napus* grown in the same soil (Van Slycken et al., 2013b).

*Z. mays* has been also widely surveyed for its potential employment in the management of HM-contaminated soil due to its rapid growth, high biomass production and general Cd-tolerance. In a field-scale trial conducted on an area of Cd-contaminated farmland in China, *Z. mays* was shown to accumulate up to 3 mg.kg^−1^ of dry biomass while maintaining grain Cd concentrations under the Chinese government's limit for coarse cereals (Xu et al., 2013). Van Slycken et al. (2013a) adopted *Z. mays* for the management of Cd, Zn, Pb and As-contaminated agricultural land. In their study, *Z. mays* was shown to remove on average 19 g Cd ha^−1^·year^−1^ and 4.3 kg Zn ha^−1^·year^−1^ with the biomass produced being comparable with *Z. mays* grown in non-contaminated soil in the same region. Moreover, the Cd content in the harvested biomass did not exceed European threshold values for animal feed. The use of biomass for biogas production was also demonstrated, with the production values being unaffected by the HM content. The issue of employing *Z. mays* for the phytoextraction of HM-contaminated soils was comprehensibly reviewed by Rizwan et al. (2017). The employment of other high-biomass-producing non-hyperaccumulating plants for trace element extraction have been also reported, both from small- and large-scale field experiments (for a comprehensive review see Vangronsveld et al., 2009).

A more advanced approach of employing plants for the clean-up of soil, proposed by Macci et al. (2013), is to take advantage of the synergic action of trees, shrubs and grasses. In their recent study, Macci et al. (2016) investigated the real-scale clean-up of a former industrial area polluted with a mixed contamination of HMs, hydrocarbons and polychlorinated biphenyls. Three different woody plants were employed, namely *Populus* sp., empress tree (*Paulownia tomentosa*), and the perennial shrub Scotch broom (*Cytisus scoparius*), in combination with naturally growing vegetation and the addition of organic matter (horse manure). Within 2 years, vegetation with any of the three species resulted in the reduction of both organic and HM contamination (average reduction of about 35, 40, and 70% in the soil content of HMs, hydrocarbons and PCBs, respectively); moreover, soil functionality was also recovered during the remediation.

### Phytoextraction employing hyperaccumulators

In a vast number of published manuscripts, hyperaccumulators have been shown to possess an exceptional ability to take up HMs from soil and store them in aerial plant organs, a trait that is seemingly promising for employment in phytoextraction. For instance, the study of Robinson et al. (1998) summarized that phytoextraction using *N. carulescens* would be entirely feasible for low Cd soil levels; since plants reached values of bioaccumulation coefficients of about 60 and 10 for 1 mg·kg^−1^ and 50 mg·kg^−1^ Cd in soil, respectively, only a single harvest would be needed to halve a soil Cd content of 10 mg·kg^−1^. Unlike Cd, the remediation of Zn was not found to be feasible within an economic time frame (i.e., < 10 years) because of the low Zn bioaccumulation coefficients (bellow 1) achieved at higher soil Zn levels. Similar conclusions were made by Nishiyama et al. (2005), whose study performed with *N. caerulescens* grown at a Cd soil concentration of 5 mg·kg^−1^ indicated that only about 2 – 6 harvests (depending on the soil type, Fluvisol or Andosol) could decrease the Cd concentration to 50%. Moreover, field trials conducted on urban HM-contaminated soil by Jacobs et al. (2017) using *N. caerulescens*, showed that Cd and Zn removal from soil was in a range that is already practically applicable for phytoextraction (Cd and Zn uptake values of 200 g.ha^−1^ and 47 kg.ha^−1^, respectively).

Other hyperaccumulating taxa have been proposed to be employable for HM removal from soils (Sarma, 2011). For instance, *B. juncea* was reported to effectively remove Cd, Cr, Cu, Ni, Pb, and Zn from a hydroponic medium (Dushenkov et al., 1995), hydrophyte water hyacinth (*Eichhornia crassipes*) was able to efficiently accumulate Cd, Cr, Cu, and Se, especially in roots. Similarly, the perennial shrub *Sesbania drummondii*, a Pb hyperaccumulator, exhibited an ability to uptake Pb (shoot concentrations of >4% Pb in hydroponic culture) while maintaining a high biomass yield (Sahi et al., 2002).

An example of an extension of hyperaccumulator-based HM phytoextraction providing an added economical value is *phytomining* (see Glossary). The field study by Robinson et al. (1997) was performed on ultramafic soils in Italy with the autochthonic Ni hyperaccumulator *Alyssum bertolonii*. The authors showed that this perennial might be used for the economically feasible phytomining of Ni, providing up to 72 kg Ni.ha^−1^ without the need to resow for a further crop. Similarly, field trials with *A. murale* recovered up to 112 kg Ni.ha^−1^ in a single crop (Robinson et al., 1997; Bani et al., 2015a,b).

### Green biotechnology application–GM plants with enhanced HM tolerance and phytoextraction efficiency

As was shown above, the use of plants might represent an economically- and ecologically-friendly alternative to conventional techniques for the decontamination/stabilization of HM-polluted sites. However, some features limit their successful use, including (i) low biomass production of many natural hyperaccumulators and (ii) low phytoextraction efficiency of plant species that, on the other hand, fulfill the required technological parameters such as high growth rate and biomass production. Therefore, the use of such a convenient plant species, but purposely genetically engineered to the “hyperaccumulator-like” phenotype (e.g., with increased HM accumulation in aboveground parts and HM tolerance), seems to be a sustainable way to utilize plants for the decontamination of polluted matrices. A general strategy for the production of such bioengineered plants has been reviewed elsewhere, for instance by Bhargava et al. (2012).

If the resultant genetically engineered plant is intended for the decontamination of HM-polluted matrices, the aim of the transgenosis is to modify the distribution of accumulated HM throughout the plant body. HM should be preferentially translocated into above-ground parts that can be easily harvested, and the plant's growth rate should not be reduced as the result of HM accumulation. Hence, sustaining root-to-shoot translocation (HM loading into xylem vessels) and subsequent vacuolization and/or sequestration in the cells of the plant aerial parts account for the most frequently employed strategies to create hyperaccumulator-like plant varieties (for more detailed reviews on the topic, see e.g., Bhargava et al., 2012; Mosa et al., 2016).

In addition to HM removal from polluted matrices, enhanced HM uptake and translocation into aboveground parts via genetic modifications has been also proposed to increase the concentration of trace elements (such as Fe, Zn, Cu, Se) in biomass for nutritional purposes when grown in non-contaminated sites (*biofortification*). For instance, transgenic wheat (*Triticum aestivum*) expresing gene *OsNAS2* encoding for nicotianamine synthase 2 from rice (*Oryza sativa*) was shown to attain dietary significant levels of Zn and Fe in grains (Singh et al., 2017). Similarly, authors Wu et al. (2018) used combination of three genes *AtNAS1* encoding for nicotianamine synthase 1 from *A. thaliana, AtFRD3* (transporter loading xylem with citrate-iron complex) and *PvFER* (ferritin from bean *Phaseolus vulgaris*) resulting in increased Zn and Fe content in grains of resultant multi-transgenic lines of *O. sativa*. A trial with *O. sativa* expressing a cisgene (see Glossary) *OsNAS2* and a transgene *SferrH-1* encoding for ferritin from soybean (*Glycine max*) proved biofortification of grains with Fe and Zn even under field conditions without any concomitant contamination with other harmful HM (Trijatmiko et al., 2016). The topic of biofortification via genetic modification was reviewed in Palmgren et al. (2008) and Borrill et al. (2014).

#### HM protein transporters

As mentioned previously, enhanced HM translocation across biological membranes most likely denotes a fundamental physiological process determining the hyperaccumulation ability of a plant. HM transport in plants includes transport through both the cytoplasmic membrane (apoplasm/symplasm interphase, e.g., uploading/unloading xylem vessels) and tonoplast and, possibly, borders of other intracellular compartments (Leitenmaier and Küpper, 2013). Alteration of these processes *via* genetic modification therefore appears to be an option to develop a plant variety suitable for HM phytoextraction and such genetic modifications have been frequently employed. Yeast cadmium factor protein YCF (a vacuolar glutathione S-conjugate pump and a member of the ATP-binding cassette transporter family associated with multi-drug resistance) is an example of a HM protein transporter used for transgenosis (Li et al., 1996). For instance, in the study of Bhuiyan et al. (2011b) the *YCF1* gene was introduced into *B. juncea* and the resultant transformants exhibited enhanced tolerance to Cd and Pb and also an increased accumulation of these HMs in shoots (about 1.5- to 2-fold compared to the wild type). The same gene was employed by Shim et al. (2013) for hybrid *Populus* sp. transgenosis. Upon Cd exposure, the resultant plants exhibited enhanced growth, reduced Cd toxicity symptoms, and increased Cd content in the aerial tissue compared to non-transgenic plants. Furthermore, the plants accumulated more Cd, Zn, and Pb in roots because they were able to establish an extensive root system in HM-contaminated mine tailing soil. Similarly, *B. juncea* plants expressing AtATM3, another mitochondrial membrane ABC transporter involved in the biogenesis of Fe-S clusters and iron homeostasis in thale cress (*Arabidopsis thaliana*), exhibited increased Cd and Pb tolerance and shoot accumulation (about 1.5- to 2.5-fold higher compared to the wild type). These changes in metallophenotype were attributed to increased expression levels of intrinsic glutathione synthetase II and phytochelatin synthase 1 induced by AtATM3 overexpression (Bhuiyan et al., 2011a).

Bacterial HM-protein transporters is another vast pool of molecules potentially employable for transgenosis in order to increase phytoextraction efficiency. Wu et al. (2011) reported ectopic expression of mutated variant of *CAX1* gene from *A. thaliana* encoding for Ca^2+^/H^+^ antiporter in petunia (*Petunia* × *hybrida*). *CAX1*-bearing plants exhibited up to 2.5-fold greater Cd tolerance and accumulation compared to the control. Furthermore, an exemplary study demonstrated that the introduction of a bacterial HM transporter PbtA into a yeast resulted in an increased accumulation of Pb, Zn, and Cd, and this transporter is further suggested to be employed for plant transgenosis aiming at increased phytoextraction efficiency and higher resistance of the plant (Suman et al., 2014). More examples of HM transporters employed in plant transgenosis are listed the Table 2.

**Table 2:** Exemplary studies dealing with GM plants with altered HM tolerance and/or accumulation ability.

**Gene or coding sequence (protein product)**	**Source**	**Recipient**	**Tested metal**	**Effect**	**References**
**TRANSGENES OF BACTERIAL ORIGIN**					
*merA* (modified) mercury(II) reductase	*Tn*21 transposon	*Nicotiana tabacum*	Hg	Enhanced mercury volatilization	He et al., 2001
*merA* (modified) mercury(II) reductase	*Tn*21 transposon	*Populus deltoides*	Hg	Enhanced mercury volatilization and tolerance	Che et al., 2003
*merA* (modified) mercury(II) reductase	*Tn*21 transposon	*Arabidopsis thaliana*	Hg (Au)	Increased Hg tolerance volatilization, increased Au tolerance	Rugh et al., 1996
*merB* (organomercurial lyase)	*Escherichia coli*	*A. thaliana*	Organomer curials	Increased tolerance to organomercurials	Bizily et al., 1999
*merC* (HM transporter), constitutive promoter	*Acidithioba-cillus ferrooxidans*	*N. tabacum, A. thaliana*	Hg	Hg hypersensitivity, enhanced Hg uptake	Sasaki et al., 2006
*merC* (HM transporter), specific membrane targeting	*Tn*21 transposon	*A. thaliana*	Cd	Increased Cd accumulation and tolerance	Kiyono et al., 2012
*merP* (Hg^2+^-binding protein)	*Bacillus megaterium*	*A. thaliana*	Hg, Cd, Pb	Enhanced Hg, Cd and Pb tolerance and accumulation	Hsieh et al., 2009
*gsh1* (γ-glutamylcysteine synthetase)	*E. coli*	*Brassica juncea*	Cd	Enhanced Cd tolerance and shoot accumulation	Zhu et al., 1999
*gsh2* (glutathione synthetase)	*E. coli*	*B. juncea*	Cd	Enhanced Cd tolerance and shoot accumulation	Liang Zhu et al., 1999
*gsh1* (γ-glutamylcysteine synthetase)	*E. coli*	*Populus canescens*	Zn	Elevated HM uptake, elevated glutathione levels	Bittsánszky et al., 2005
		hybrid *Populus tremula × Populus alba)*	Cd	Enhanced Cd accumulation in young leaves	Koprivova et al., 2002
*gsh1* (γ-glutamylcysteine synthetase) and *gsh2* (glutathion synthetase)	*E. coli*	*A. thaliana*, co-expression	Hg, As	Enhanced Hg and As tolerance, enhanced Hg accumulation in aboveground tissues	Li et al., 2006b
*GCS* and *GS* (γ-glutamylcysteine synthetase and glutathione synthetase)	*Streptococcus thermophilus*	*Beta vulgaris*	Cd, Zn, Cu	Enhanced Cd, Zn and Cu tolerance and accumulation; increased multiple HM tolerance and accumulation without selectivity among Cd, Zn and Cu	Liu et al., 2015
*zntA* (Pb^2+^/Zn^2+^/Cd^2+^-transporting P-ATPase)	*E. coli*	*A. thaliana*	Pb, Cd	Enhanced Pb and Cd tolerance, diminished Cd and Pb shoot accumulation	Lee et al., 2003
1-aminocyclo-propane-1-carboxylic acid (ACC) deaminase	*Enterobacter cloacae*	*Lycopersicon aesculentum*	Cd, Co, Cu, Ni, Pb, Zn	Enhanced tolerance to and altered accumulation of Cd, Co, Cu, Ni, Pb and Zn	Grichko et al., 2000
ACC deaminase, *iaa* (tryptophan monooxygenase)	*Pseudomonas putida* (ACC deaminase), *Agrobacte-rium tumefaciens* (*iaa*)	*N. tabacum, Petunia × hybrida* co-expression	Cu, Co	Increased Cu and Co tolerance and accumulation, growth in a complex contaminated soil	Zhang et al., 2008
*arsC* (arsenate reductase)	*E. coli*	*N. tabacum, A. thaliana*	Cd	Enhanced Cd tolerance and shoot accumulation	Dhankher et al., 2003
**TRANSGENES OF FUNGAL ORIGIN**					
*CUP1* (metallothionein)	*Saccharomyces cerevisiae*	*N. tabacum*	Cd	Increased Cd tolerance, increased Cd levels in roots whereas decreased in shoots	Krystofova et al., 2012
	*Saccharomyces cerevisiae*	*N. tabacum*	Cu	Enhanced Cu accumulation, no Cd-associated phenotypical changes	Thomas et al., 2003
*YCF1* (ATP-binding cassette transporter)	*S. cerevisiae*	*B. juncea*	Cd, Pb	Enhanced Cd and Pb tolerance and shoot accumulation	Bhuiyan et al., 2011b
*tzn1* (high specificity zn transporter)	*Neurospora crassa*	*N. tabacum*	Zn	Enhanced Zn accumulation	Dixit et al., 2010
**TRANSGENES OF PLANT ORIGIN**					
*MuSI* (multiple stress response gene I)	*Ipomoea batatas*	*N. tabacum*	Cd	Enhanced Cd tolerance, diminished Cd accumulation and root-to-shoot translocation	Kim et al., 2011
*PCS1* (phytochelatin synthase)	*Thlaspi arvense*	*Nicotiana glauca*	Cd, Zn, Pb, Cu	Enhanced HM accumulation, high biomass production in real mining soil experiment	Martínez et al., 2006
	*Ceratophyllum demersum*	*N. tabacum*	Cd, As	Enhanced Cd and As root accumulation	Shukla et al., 2012
	*Nelumbo nucifera*	*A. thaliana*	Cd	Enhanced Cd accumulation	Liu et al., 2012
	*Cynodon dactylon*	*N. tabacum*	Cd	Increase Cd tolerance and accumulation	Li et al., 2006a
*PCS1* (phytochelatin synthase) and *GSH1*	*Allium sativum* (*PCS1*), *S. cerevisiae* (*GSH1*)	*A. thaliana*, co-expression	Cd, As	Enhanced Cd and As tolerance and shoot accumulation	Guo et al., 2008
*GS* cysteine synthase	*Spinacia oleracea*	*N. tabacum*	Cd, Se, Ni	Increased Cd, Se and Ni tolerance, enhanced Cd shoot accumulation	Kawashima et al., 2004
*GCS* (γ-glutamylcysteine synthetase)	*Phragmites australis*	*Agrostis stolonifera*	Cd	Increased Cd tolerance and accumulation	Zhao et al., 2010
*CBP4* (calmodulin-binding channel-like protein)	*N. tabacum*	*N. tabacum* overexpression	Ni, Pb	Increased Ni tolerance Pb and hypersensitivity, reduced Ni and enhanced Pb accumulation	Arazi et al., 1999
*MRP7* (ATP-binding cassette transporter)	*A. thaliana*	*N. tabacum*	Cd	Increased Cd tolerance, decreased Cd root-to-shoot translocation, enhanced Cd accumulation in leaf vacuoles	Wojas et al., 2009
*ATM3* (ATP-binding cassette transporter)	*A. thaliana*	*B. juncea*	Cd, Pb	Increased Cd and Pb tolerance and accumulation	Bhuiyan et al., 2011a
*CAX1* (mutated variant) (cation/H^+^ antiporter)	*A. thaliana*	*Petunia × hybrida*	Cd	Increased Cd tolerance and accumulation,	Wu et al., 2011
*MT2b* (metallothionein) and *HMA4* (P_1B_-ATPase)	*A. thaliana*	*N. tabacum*, co-expression	Cd, Zn	Enhanced Cd tolerance, enhanced Cd and Zn root-to-shoot transport, synergy between the two transgenes	Grispen et al., 2011
*MT2b* (metallothionein)	*A. sativum*	*A. thaliana*	Cd	Enhanced Cd tolerance and accumulation	Zhang et al., 2006
*CET3, CET4* (cation-efflux transporters)	*B. juncea*	*N. tabacum*, co-expression	Cd	Enhanced Cd tolerance, altered Cd accumulation	Lang et al., 2011
*ACBP1* (acylCoA-binding protein)	*A. thaliana*	*A. thaliana* overexpression	Pb	Enhanced Pb tolerance and shoot accumulation	Xiao et al., 2008
**TRANSGENES OF OTHER ORIGIN**					
*MTII* (clas II metallothionein)	*Homo sapiens sapiens*	*N. tabacum*	Cd	Decreased Cd levels in leaves whereas increased in roots and stems	de Borne et al., 1998
Pseudophytochelatin Met(GluCys)_6_Gly	Synthetic	*N. tabacum*	Cd	Enhanced Cd tolerance and shoot accumulation	Postrigan et al., 2012

#### Protein/peptidic HM chelators and low-molecular binding agents

Another strategy adopted to artificially enhance the HM accumulation capacity and tolerance of plants is through increased levels of HM binding agents *in planta*. Generally, it is hypothesized that the cell capacity to accumulate HM should be enhanced via the increased intracellular production of HM-ions binding sites, along with increasing metalloresistance through avoiding the appearance of “free” metal ions in the plant. Several examples of specific genetic determinants introduced into various plant species with the aim of increasing intracellular metal-chelating compounds can be found in Table 2. A frequently investigated approach is exemplified by the overproduction of peptidic chelators such as glutathione (GSH) and phytochelatins (PC) through the overexpression of the enzymes of their biosynthetic pathways. An example of an effort to increase intracellular GSH content is the overexpression of genes encoding for γ-glutamylcysteine synthetase (γ-ECS) and cysteine synthetase (GS), of both bacterial and eukaryotic origin (Liang Zhu et al., 1999; Koprivova et al., 2002; Bittsánszky et al., 2005; Li et al., 2006b; Zhao et al., 2010). These enzymes catalyze two ATP-dependent reactions: the formation of γ-glutamylcysteine (γ-ES) from glutamate and cysteine and the addition of glycine at the C-terminal end of γ-ES, respectively, yielding GSH (Meister, 1988). For instance, in the pioneering work of Noctor et al. (1996), transgenic *Populus* sp. bearing γ-ECS (*gsh1*) from *E. coli* were generated. Foliar γ-ES and glutathione content increased 10- and 3-fold, respectively, without affecting either the general physiological parameters of the transgenic plants or the reduction state of the glutathione pool and the foliar cysteine content. Several other studies were published employing genes for either γ-ECS or GS, or both simultaneously, for the alteration of the metallophenotype of plant species such as *B. juncea* (Liang Zhu et al., 1999; Zhu et al., 1999), *Populus* sp. (Koprivova et al., 2002; Bittsánszky et al., 2005), *A. thaliana* (Li et al., 2006b), creeping bentgrass (*Agrostis palustris*) (Zhao et al., 2010), etc. The resultant (double-)transgenic plants generally exhibited an increased rate of tolerance and accumulation of HMs such as Cd (Liang Zhu et al., 1999; Zhu et al., 1999; Koprivova et al., 2002; Zhao et al., 2010), Zn (Bittsánszky et al., 2005), or Hg (Li et al., 2006b).

A similar approach to increase the pool of HM-binding thiol compounds is the introduction of phytochelatin synthase (PCS), an enzyme catalyzing the formation of PC from the precursor γ-ES (Ha et al., 1999; Clemens and Persoh, 2009). PCS-encoding genes predominantly of plant origin were used for the transgenosis, leading to enhanced HM accumulation and tolerance (Table 2; Li et al., 2006a; Martínez et al., 2006; Guo et al., 2008; Liu et al., 2012; Shukla et al., 2012).

Unlike GSH and PCs, metallothioneins (MT) are gene-encoded metal binding proteins with multiple functions *in planta*, including metal homeostasis (for a comprehensive review, see Leszczyszyn et al., 2013). These small proteins have been found to exert HM binding capacity *in vivo* including Zn, Cd and Cu, when heterologously and ectopically expressed (Kille et al., 1991; Tommey et al., 1991; Evans et al., 1992; Abdullah et al., 2002). In addition to the inspection of their physiological function, a possible use of MT for enhancing metalloresistance and/or the accumulation capacity of GM plants was proposed by several authors (Thomas et al., 2003; Zhang et al., 2006; Krystofova et al., 2012). Nevertheless, the effect of such ectopic and heterologous expression on the metallophenotype is rather difficult to predict; accumulation and tolerance patterns differ among metal elements and across studies (for instance Krystofova et al., 2012 vs. Thomas et al., 2003, Table 2). Since in the majority of studies MT imparted an increased metallotolerance to the resultant transgenic plants together with an enhanced retention of the studied HM in roots, MT are more likely to be employed in protecting plants against HM stress in contaminated locations rather than for *metallocrop* preparation (Krystofova et al., 2012).

A study by Pianelli et al. (2005) investigated another metal ligand molecule, nicotianamine, an ubiquitous non-proteinogenic amino acid biosynthesized from methionine, involved in plant Fe homeostasis and able to bind various other metals as well (Rudolph et al., 1985). *A. thaliana* plants overexpressing nicotianamine synthase cDNA (*NAS1*) from the *N. caerulescens* exhibited an up to 100-fold higher accumulation of nicotianamine in their tissue and higher Ni tolerance and accumulation than the control. The authors proposed this gene to be introduced into suitable high-biomass plant species in combination with a HM-complex root-to-shoot translocator (e.g., yellow stripe 1, *YSL1*; Curie et al., 2001) in order to enhance the root-to-shoot ratio of accumulated metal and therefore “move” the metal to harvestable parts.

#### “gene stacking” strategy

The term “gene stacking” (or “gene pyramiding”) refers to the simultaneous expression or manipulation of multiple transgenes in a single GM plant or hybrid variety (Halpin, 2005). The accumulation of traits leading to an increased HM uptake and storage in harvestable parts and/or increased metalloresistance represents an advanced approach to modifying the plant metallophenotype for use in HM phytoextraction. In the study by Li et al. (2006b), *A. thaliana* hybrid plants of two single-transgenic lines bearing γ-ECS and GS were prepared, and their metallophenotype was compared to the parental one. Enhanced PC levels in roots as well as increased Hg tolerance and accumulation was observed for the ECS/GS co-expressing lines achieving a Hg bioconcentration factor as high as 600. Similar findings by Guo et al. (2008) showed that dual-transgenic *A. thaliana* plants overexpressing both PCS and γ-ECS (derived from garlic and yeasts, respectively) led to elevated PC levels and exhibited significantly higher Cd/As tolerance and accumulation than single transgenic and non-transgenic controls (about 2.5 times and 10 times higher for the two elements, respectively).

Synergy in the function of two genes expressed simultaneously was also shown in the work of Grispen et al. (2011). *A. thaliana* genes *AtMT2b* and *AtHMA4* encoding for a IIb-class metallothionein and a stellar Zn^2+^- and Cd^2+^-transporting ATPase, respectively, were introduced into *N. tabacum* plants, and the double transformants exhibited enhanced Cd tolerance (ca 2-fold), enhanced Cd/Zn root-to-shoot transport, but unaltered Zn tolerance and overall Cd/Zn uptake, compared with both non-transgenic and single-transgenic controls. Hence, the authors highlighted the synergy between two genes introduced into plants. Since the observed metallophenotype was altered only moderately, they suggested (especially for the *HMA4* gene) the use of the *A. thaliana* native promoter of the *HMA4* gene instead of the constitutive cauliflower mosaic virus promoter and, possibly, the introduction of multiple transgene copies. Both proposed modifications should imitate the intrinsic tissue/organ localization and regulation of those found in the natural hyperaccumulator rockcress *Arabidopsis halleri* (Hanikenne et al., 2008).

#### Phytoextraction of HM using GM plants in the field?

Several articles comprehensively reviewing the issue of the potential employment of GM plants in HM phytoremediation have been released over the last decade (Cherian and Oliveira, 2005; Macek et al., 2008, 2009, 2013; Kotrba et al., 2009; Sarma, 2011; Bhargava et al., 2012; Ibañez et al., 2016). A number of various transgenes of both bacterial and eukaryotic origin have been employed (examples are listed in Table 2), predominantly in lab-scale studies on model plant species such as *N. tabacum, A. thaliana*, or tomato (*Lycopersicon esculentum*). In the majority of published studies, HM removal and tolerance experiments were only performed under model controlled conditions, typically in “ideal” agar-based or hydroponic cultures with only one metal element being examined in a single experiment; only a few studies were performed on a mixture of different metal elements, investigating interactions between the metals (e.g., Liu et al., 2015). Moreover, only several studies have moved from model conditions to pot-scale experiments, some of which were performed in real HM-contaminated soil (Bennett et al., 2003; Heaton et al., 2003; Pavlikova et al., 2004; Martínez et al., 2006; Rodriguez et al., 2008; Pilon-Smits and Leduc, 2009; Shim et al., 2013). Nevertheless, the “giant leap for mankind,” i.e., remediation experiments performed under field conditions, was shown to be indispensable for the reliable assessment of the applicability of GM plants for phytoremediation. The effect of examined genetic determinants on the phenotype can be severely influenced by real conditions *in situ*, including the co-occurrence of other organic and inorganic contaminants as many different contaminating metal elements tend to occur simultaneously (Banerjee et al., 1999), the bioavailability of micro- and macronutrients, soil type, content of organic matter, climatic conditions, interactions with other (micro)organisms, etc.

Among the few published studies that meet the requirement of *in situ* testing, Bañuelos et al. (2005) showed that GM plants can perform efficiently under field conditions. In their work, three different transgenic lines of *B. juncea* plants were planted in a field plot with a layer of Se, B and salt contaminated sediment covered with a layer of non-contaminated nutrient-rich soil and cultivated under continuous irrigation for 45 days. Even after this relatively short time period, plants bearing genes encoding for adenosine triphosphate sulfurylase (APS), γ-ECS and GS were able to accumulate 4.3-, 2.8-, and 2.3-fold more Se, respectively, in their shoots than the control, with the first value theoretically corresponding to 4.4% of the total extractable Se present in the contaminated drainage sediment. The aerial biomass produced was significantly lower than with control plants cultivated in the pristine soil (about 80% for GS plants), but still about 50% higher than for the wild-type strain kept in the polluted soil. Even though the duration of this field trial was short, the results indicated the potential of such GM plants for Se-phytoremediation.

The aforementioned work of Shim et al. (2013) was another study performed under real field conditions. It assessed the phytoextraction potential of transgenic *Populus* sp. plants hybrids of *P. alba* × *P. tremula* var. *glandulosa* BH1 (non-flowering mutant clone) harboring the yeast HM transporter gene *Ycf1*. During the field experiment, pre-cultivated cuttings of *Ycf1*-plants were kept in pots containing diluted Zn mine tailing soil under the open sky for a period of over 6 months. During this period, *Ycf1*-plants accumulated approximately 4–6 times more Cd in their shoots than non-GM control plants, while maintaining the same biomass production. The root system of GM plants was even more developed. Although these findings looked promising in terms of the use of the *Ycf1*-plants for the removal of Cd, Zn, As, and Pb from soils, the authors did not support this idea. The bioconcentration factor of the *Ycf* 1-plants of *Populus* sp. under field conditions was calculated to be only 0.3 for Cd, 0.9 for Zn, 0.2 for As, and 1.9 for Pb, values in strong contrast with those found for the hydroponic culture also surveyed in the study. The minimal bioconcentration factor value essential for effective short-term remediation (i.e., in time-span of ca 10 crops) using these specific *Ycf1*-bearing plants was estimated to be around 14. Nevertheless, the authors proposed the possible application of those GM plants for long-term phytoextraction with concurrent phytostabilization of polluted sites. The entire study was an example of a possible discrepancy between results obtained under hydroponic and real soil conditions, which needs to be considered before making conclusions about the potential applicability of any “GM plants with enhanced phytoextraction efficiency.” On the other hand, the field trial carried out by Bañuelos et al. (2005) with GM *B. juncea* described above basically confirmed the data obtained in their previous studies performed under model conditions (Liang Zhu et al., 1999; Pilon-Smits et al., 1999; Zhu et al., 1999).

## Conclusions and future perspectives

The first vector and bacterial strain system that enabled the introduction of genetic modifications into plants emerged in 1985 (Deblaere et al., 1985). Over the next three decades, the process of transgenosis has become routinely used for the generation of GM plants with many different goals. The methods of genetic engineering have provided us with a powerful tool for producing a vast number of model GM plants, thereby enormously contributing to our understanding of HM homeostasis and detoxification, ROS-defense systems, etc.

The possibility of employing GM plants for the clean-up of HM-contaminated matrices has been intensively researched, a number of plant taxa bearing transgenes of various origins and presumptive functions have been surveyed. As summarized herein, many studies proved that the resultant transgenic plants manifested metallophenotypes making such an approach promising for use in phytoextraction. Nevertheless, the actual use of GM plants for soil clean-up has been relatively limited. Only several published GM varieties fulfill the requirements for phytoextraction efficiency that would allow the clean-up process to be feasible in terms of time and costs.

However, GM varieties have been so far almost solely prepared for research purposes that examine the usability of a genetic determinant, incorporating no more than pot-scale or controlled field experiments. As a result, their direct introduction into the environment is rather controversial, since they do not meet the requirements for modern GM organisms, i.e., the absence of a selection marker gene and vector backbone, the site-specific character of genetic modification, the use of genes from an evolutionary close gene pool (see Glossary for cisgenesis or intragenesis), etc. Further improvements are required in the genesis of new GM varieties, which would allow both the development of plants with superior metallophenotypes, and their commercialization even in countries where GM organisms are poorly accepted (Kamthan et al., 2016). Recent advances in the genome editing system CRISPR/Cas9 have enabled highly site-specific modifications of many plant genomes, and has therefore become a powerful tool for the development of selectable-marker-free transgenic hyperaccumulators potentially more suitable for real conditions (for recent reviews, see Bortesi and Fischer, 2015; Liu et al., 2017).

At the same time, a more thorough assessment of the risks related to the introduction of GM plants into the environment is indispensable prior to their use in the field (Hilbeck et al., 2011; Sanvido et al., 2012). With GM plants intended for phytoextraction, such risks include gene-flow from transgenics which could quantitatively reduce the genetic diversity of both wild and domesticated relatives, the horizontal transfer of transgenes into non-relative organisms including prokaryotes e.g., through cross-pollination, *Agrobacterium*-mediated transformation with naked DNA, etc. (EFSA Panel On Geneticlay Modified Organisms (GMO), 2010; Tsatsakis et al., 2017). We also propose another environmental risk to be considered prior to field applications of both natural and transgenic hyperaccumulators–the toxicity of HM-enriched biomass and subsequent impact on wildlife *in situ*, including herbivores, natural enemies, pollinators, decomposers, microbial symbionts, and overall biodiversity.

Finally, as shown above in this article, recent field studies have demonstrated that some hyperaccumulators meet the requirements for utilization in a real phytoextraction process, especially for their enormous HM bioaccumulation rates being far higher than those of high-biomass non-hyperaccumulators including those from genus *Salix, Populus*, etc. With more effort put into more modern GM techniques, the performance of many plant taxa can be further improved and efficiently used for large-scale phytoextraction purposes in real locations.

## Author contributions

All authors listed have made a substantial, direct and intellectual contribution to the work, and approved it for publication.

### Conflict of interest statement

The authors declare that the research was conducted in the absence of any commercial or financial relationships that could be construed as a potential conflict of interest.

## References

[B1] Abdullah S. N. A. Cheah S. C. Murphy D. J. (2002). Isolation and characterisation of two divergent type 3 metallothioneins from oil palm, Elaeis guineensis. Plant Physiol. Biochem. 40, 255–263. 10.1016/S0981-9428(02)01366-9

[B2] Ali H. Khan E. (2018). What are heavy metals? Long-standing controversy over the scientific use of the term ‘heavy metals' – proposal of a comprehensive definition. Toxicol. Environ. Chem. 100, 6–19. 10.1080/02772248.2017.1413652

[B3] Arazi T. Sunkar R. Kaplan B. Fromm H. (1999). A tobacco plasma membrane calmodulin-binding transporter confers Ni2+ tolerance and Pb2+ hypersensitivity in transgenic plants. Plant J. 20, 171–182. 10.1046/j.1365-313x.1999.00588.x 10571877

[B4] Arena C. Figlioli F. Sorrentino M. C. Izzo L. G. Capozzi F. Giordano S.. (2017). Ultrastructural, protein and photosynthetic alterations induced by Pb and Cd in *Cynara cardunculus L*., and its potential for phytoremediation. Ecotoxicol. Environ. Saf. 145, 83–89. 10.1016/j.ecoenv.2017.07.015 28708985

[B5] Assuncao A. G. L. Bleeker P. ten Bookum W. M. Vooijs R. Schat H. (2008). Intraspecific variation of metal preference patterns for hyperaccumulation in *Thlaspi caerulescens*: evidence from binary metal exposures. Plant Soil 303, 289–299. 10.1007/s11104-007-9508-x

[B6] Baker A. J. M. (1981). Accumulators and excluders-strategies in the response of plants to heavy-metals. J. Plant Nutr. 3, 643–654. 10.1080/01904168109362867

[B7] Baker A. J. M. Brooks R. R. (1989). Terrestrial higher plants which hyperaccumulate metallic elements-a review of their distribution, ecology and phytochemistry. Biorecovery 1, 81–126.

[B8] Baker A. J. M. McGrath S. P. Sidoli C. M. D. Reeves R. D. (1994). The possibility of in situ heavy metal decontamination of polluted soils using crops of metal-accumulating plants. Resour. Conserv. Recycl. 11, 41–49. 10.1016/0921-3449(94)90077-9

[B9] Banerjee K. Helwick R. P. Gupta S. (1999). A treatment process for removal of mixed inorganic and organic arsenic species from groundwater. Environ. Progr. 18, 280–284. 10.1002/ep.670180415

[B10] Bani A. Echevarria G. Sulce S. Morel J. L. (2015a). Improving the agronomy of alyssum murale for extensive phytomining: a five-year field study. Int. J. Phytoremed. 17 (1-6), 117–127. 10.1080/15226514.2013.862204 25237722

[B11] Bani A. Echevarria G. Zhang X. Benizri E. Laubie B. Morel J. L. (2015b). The effect of plant density in nickel-phytomining field experiments with *Alyssum murale* in Albania. Austral. J. Botany 63, 72–77. 10.1071/BT14285

[B12] Bañuelos G. Terry N. Leduc D. L. Pilon-Smits E. A. H. Mackey B. (2005). Field trial of transgenic Indian mustard plants shows enhanced phytoremediation of selenium-contaminated sediment. Environ. Sci. Technol. 39, 1771–1777. 10.1021/es049035f 15819237

[B13] Benáková M. Ahmadi H. Ducaiova Z. Tylova E. Clemens S. Tuma J. (2017). Effects of Cd and Zn on physiological and anatomical properties of hydroponically grown Brassica napus plants. Environ. Sci. Pollution Res. 24, 20705–20716. 10.1007/s11356-017-9697-7 28714046

[B14] Bennett L. E. Burkhead J. L. Hale K. L. Terry N. Pilon M. Pilon-Smits E. A. H. (2003). Analysis of transgenic Indian mustard plants for phytoremediation of metal-contaminated mine tailings. J. Environ. Qual. 32, 432–440. 10.2134/jeq2003.4320 12708665

[B15] Bhargava A. Carmona F. F. Bhargava M. Srivastava S. (2012). Approaches for enhanced phytoextraction of heavy metals. J. Environ. Manage. 105, 103–120. 10.1016/j.jenvman.2012.04.002 22542973

[B16] Bhuiyan M. S. U. Min S. R. Jeong W. J. Sultana S. Choi K. S. Lee Y. (2011a). Overexpression of AtATM3 in *Brassica juncea* confers enhanced heavy metal tolerance and accumulation. Plant Cell Tissue Organ Culture 107, 69–77. 10.1007/s11240-011-9958-y

[B17] Bhuiyan M. S. U. Min S. R. Jeong W. J. Sultana S. Choi K. S. Song W. Y. (2011b). Overexpression of a yeast cadmium factor 1 (YCF1) enhances heavy metal tolerance and accumulation in *Brassica juncea*. Plant Cell Tissue Organ Culture 105, 85–91. 10.1007/s11240-010-9845-y

[B18] Bittsánszky A. Kömives T. Gullner G. Gyulai G. Kiss J. Heszky L.. (2005). Ability of transgenic poplars with elevated glutathione content to tolerate zinc(2^+^) stress. Environ. Int. 31, 251–254. 10.1016/j.envint.2004.10.001 15661291

[B19] Bizily S. P. Rugh C. L. Summers A. O. Meagher R. B. (1999). Phytoremediation of methylmercury pollution: merB expression in *Arabidopsis thaliana* confers resistance to organomercurials. Proc. Natl. Acad. Sci. U.S.A. 96, 6808–6813. 10.1073/pnas.96.12.6808 10359794 PMC21997

[B20] Borrill P. Connorton J. M. Balk J. Miller A. J. Sanders D. Uauy C. (2014). Biofortification of wheat grain with iron and zinc: integrating novel genomic resources and knowledge from model crops. Front. Plant Sci. 5:53. 10.3389/fpls.2014.00053 24600464 PMC3930855

[B21] Bortesi L. Fischer R. (2015). The CRISPR/Cas9 system for plant genome editing and beyond. Biotechnol. Adv. 33, 41–52. 10.1016/j.biotechadv.2014.12.006 25536441

[B22] Brooks R. R. Chambers M. F. Nicks L. J. Robinson B. H. (1998). Phytomining. Trends Plant Sci. 3, 359–362. 10.1016/S1360-1385(98)01283-7

[B23] Brooks R. R. Morrison R. S. Reeves R. D. Dudley T. R. Akman Y. (1979). Hyper-accumulation of nickel by *Alyssum linnaeus* (Cruciferae). Proc. R. Soc. B Biol. Sci. 203, 387–403. 10.1098/rspb.1979.0005 34161

[B24] Brooks R. R. Radford C. C. (1978). Nickel accumulation by European species of genus *Alyssum*. Proc. R. Soc. B Biol. Sci. 200, 217–224. 10.1098/rspb.1978.0016

[B25] Chandra R. Kang H. (2016). Mixed heavy metal stress on photosynthesis, transpiration rate, and chlorophyll content in poplar hybrids. Forest Sci. Technol. 12, 55–61. 10.1080/21580103.2015.1044024

[B26] Chaney R. L. Malik M. Li Y. M. Brown S. L. Brewer E. P. Angle J. S.. (1997). Phytoremediation of soil metals. Curr. Opin. Biotechnol. 8, 279–284. 10.1016/S0958-1669(97)80004-3 9206007

[B27] Chapman P. M. Thornton I. Persoone G. Janssen C. Godtfredsen K. Z'Graggen M. N. (1996). International harmonization related to persistence and bioavailability. Human Ecol. Risk Assess. An Int. J. 2, 393–404. 10.1080/10807039609383618

[B28] Che D. Meagher R. B. Heaton A. C. P. Lima A. Rugh C. L. Merkle S. A. (2003). Expression of mercuric ion reductase in Eastern cottonwood (*Populus deltoides*) confers mercuric ion reduction and resistance. Plant Biotechnol. J. 1, 311–319. 10.1046/j.1467-7652.2003.00031.x 17163907

[B29] Cherian S. Oliveira M. M. (2005). Transgenic plants in phytoremediation: recent advances and new possibilities. Environ. Sci. Technol. 39, 9377–9390. 10.1021/es051134l 16475312

[B30] Clemens S. Persoh D. (2009). Multi-tasking phytochelatin synthases. Plant Sci. 177, 266–271. 10.1016/j.plantsci.2009.06.008

[B31] Cristaldi A. Conti G. O. Jho E. H. Zuccarello P. Grasso A. Copat C. (2017). Phytoremediation of contaminated soils by heavy metals and PAHs. A brief review. Environ. Technol. Innov. 8, 309–326. 10.1016/j.eti.2017.08.002

[B32] Cunningham S. D. Berti W. R. Huang J. W. W. (1995). Phytoremediation of contaminated soils. Trends Biotechnol. 13, 393–397. 10.1016/S0167-7799(00)88987-8

[B33] Cunningham S. D. Ow D. W. (1996). Promises and prospects of phytoremediation. Plant Physiol. 110, 715–719. 10.1104/pp.110.3.715 12226213 PMC157769

[B34] Curie C. Panaviene Z. Loulergue C. Dellaporta S. L. Briat J.-F. Walker E. L. (2001). Maize yellow stripe1 encodes a membrane protein directly involved in Fe(III) uptake. Nature 409, 346–349. 10.1038/35053080 11201743

[B35] de Borne F. D. Elmayan T. de Roton C. de Hys L. Tepfer M. (1998). Cadmium partitioning in transgenic tobacco plants expressing a mammalian metallothionein gene. Molecul. Breed. 4, 83–90. 10.1023/A:1009669412489

[B36] Deblaere R. Bytebier B. De Greve H. Deboeck F. Schell J. Van Montagu M.. (1985). Efficient octopine Ti plasmid-derived vectors for Agrobacterium-mediated gene transfer to plants. Nucleic Acids Res. 13, 4777–4788. 10.1093/nar/13.13.4777 4022773 PMC321826

[B37] Dhankher O. P. Shasti N. A. Rosen B. P. Fuhrmann M. Meagher R. B. (2003). Increased cadmium tolerance and accumulation by plants expressing bacterial arsenate reductase. N. Phytol. 159, 431–441. 10.1046/j.1469-8137.2003.00827.x 33873364

[B38] Dixit P. Singh S. Vancheeswaran R. Patnala K. Eapen S. (2010). Expression of a *Neurospora crassa* zinc transporter gene in transgenic *Nicotiana tabacum* enhances plant zinc accumulation without co-transport of cadmium. Plant Cell Environ. 33, 1697–1707. 10.1111/j.1365-3040.2010.02174.x 20492552

[B39] Dushenkov V. Kumar P. Motto H. Raskin I. (1995). Rhizofiltration-the use of plants to remove heavy-metals from aqueous streams. Environ. Sci. Technol. 29, 1239–1245. 10.1021/es00005a015 22192017

[B40] EFSA Panel On Geneticlay Modified Organisms (GMO) (2010). Guidance on the environmental risk assessment of genetically modified plants. EFSA J. 8:1879 10.2903/j.efsa.2010.1879 PMC13129916 42079552

[B41] Einspahr D. W. (1976a). Influence of short-rotation forestry and paper quality. I, Short-rotation conifers. Tappi 59, 53–56.

[B42] Einspahr D. W. (1976b). Influence of short-rotation forestry on pulp and paper quality. II, Short-rotation hardwood. Tappi 59, 63–66.

[B43] Evans K. M. Gatehouse J. A. Lindsay W. P. Shi J. Tommey A. M. Robinson N. J. (1992). Expression of the pea metallothionein-like gene PsMTA in *Escherichia coli* and *Arabidopsis thaliana* and analysis of trace metal ion accumulation: implications for PsMTA function. Plant Mol. Biol. 20, 1019–1028. 10.1007/BF00028889 1463837

[B44] Gomes M. P. Marques T. C. L. L. S. M. Carneiro M. M. L. C. Soares Â. M. (2012). Anatomical characteristics and nutrient uptake and distribution associated with the Cd-phytoremediation capacity of *Eucalyptus camaldulenses* Dehnh. J. Soil Sci. Plant Nutr. 12, 481–496. 10.4067/S0718-95162012005000010

[B45] Greger M. Landberg T. (1999). Use of willow in phytoextraction. Int. J. Phytoremediation 1, 115–123. 10.1080/15226519908500010 25955021

[B46] Grichko V. P. Filby B. Glick B. R. (2000). Increased ability of transgenic plants expressing the bacterial enzyme ACC deaminase to accumulate Cd, Co, Cu, Ni, Pb, and Zn. J. Biotechnol. 81, 45–53. 10.1016/S0168-1656(00)00270-4 10936659

[B47] Grispen V. M. J. Hakvoort H. W. J. Bliek T. Verkleij J. A. C. Schat H. (2011). Combined expression of the *Arabidopsis* metallothionein MT2b and the heavy metal transporting ATPase HMA4 enhances cadmium tolerance and the root to shoot translocation of cadmium and zinc in tobacco. Environ. Exp. Bot. 72, 71–76. 10.1016/j.envexpbot.2010.01.005

[B48] Guo J. Dai X. Xu W. Ma M. (2008). Overexpressing GSH1 and AsPCS1 simultaneously increases the tolerance and accumulation of cadmium and arsenic in *Arabidopsis thaliana*. Chemosphere 72, 1020–1026. 10.1016/j.chemosphere.2008.04.018 18504054

[B49] Ha S. B. Smith A. P. Howden R. Dietrich W. M. Bugg S. O'Connell M. J. (1999). Phytochelatin synthase genes from *Arabidopsis* and the yeast *Schizosaccharomyces pombe*. Plant Cell 11, 1153–1164. 10.1105/tpc.11.6.1153 10368185 PMC144235

[B50] Halpin C. (2005). Gene stacking in transgenic plants–the challenge for 21st century plant biotechnology. Plant Biotechnol. J. 3, 141–155. 10.1111/j.1467-7652.2004.00113.x 17173615

[B51] Hanikenne M. Talke I. N. Haydon M. J. Lanz C. Nolte A. Motte P.. (2008). Evolution of metal hyperaccumulation required cis-regulatory changes and triplication of HMA4. Nature 453, 391–395. 10.1038/nature06877 18425111

[B52] He Y. K. Sun J. G. Feng X. Z. Czako M. Marton L. (2001). Differential mercury volatilization by tobacco organs expressing a modified bacterial merA gene. Cell Res. 11, 231–236. 10.1038/sj.cr.7290091 11642409

[B53] Heaton A. C. P. Rugh C. L. Kim T. Wang N. J. Meagher R. B. (2003). Toward detoxifying mercury-polluted aquatic sediments with rice genetically engineered for mercury resistance. Environ. Toxicol. Chem. 22, 2940–2947. 10.1897/02-442 14713034

[B54] Hilbeck A. Meier M. Römbke J. Jänsch S. Teichmann H. Tappeser B. (2011). Environmental risk assessment of genetically modified plants-concepts and controversies. Environ. Sci. Eur. 23:13 10.1186/2190-4715-23-13 19694493

[B55] Hinchee M. Rottmann W. Mullinax L. Zhang C. S. Chang S. J. Cunningham M.. (2009). Short-rotation woody crops for bioenergy and biofuels applications. In Vitro Cell. Dev. Biol. Plant 45, 619–629. 10.1007/s11627-009-9235-5 19936031 PMC2778772

[B56] Hsieh J. L. Chen C. Y. Chiu M. H. Chein M. F. Chang J. S. Endo G.. (2009). Expressing a bacterial mercuric ion binding protein in plant for phytoremediation of heavy metals. J. Hazard. Mater. 161, 920–925. 10.1016/j.jhazmat.2008.04.079 18538925

[B57] Ibañez S. Talano M. Ontanon O. Suman J. Medina M. I. Macek T.. (2016). Transgenic plants and hairy roots: exploiting the potential of plant species to remediate contaminants. N. Biotechnol. 33, 625–635. 10.1016/j.nbt.2015.11.008 26703807

[B58] Ivanova L. A. Ronzhina D. A. Ivanov L. A. Stroukova L. V. Peuke A. D. Rennenberg H. (2011). Over-expression of gsh1 in the cytosol affects the photosynthetic apparatus and improves the performance of transgenic poplars on heavy metal-contaminated soil. Plant Biol. 13, 649–659. 10.1111/j.1438-8677.2010.00422.x 21668606

[B59] Jacobs A. Drouet T. Sterckeman T. Noret N. (2017). Phytoremediation of urban soils contaminated with trace metals using *Noccaea caerulescens*: comparing non-metallicolous populations to the metallicolous ‘Ganges' in field trials. Environ. Sci. Pollut. Res. Int. 24, 8176–8188. 10.1007/s11356-017-8504-9 28144868

[B60] Jacobsen E. Schouten H. J. (2009). Cisgenesis: an important sub-invention for traditional plant breeding companies. Euphytica 170:235 10.1007/s10681-009-0037-y

[B61] Jaffré T. Brooks R. R. Lee J. Reeves R. D. (1976). *Sebertia acuminata*: a hyperaccumulator of nickel from New Caledonia. Science 193, 579–580. 17759588 10.1126/science.193.4253.579

[B62] Kamthan A. Chaudhuri A. Kamthan M. Datta A. (2016). Genetically modified (GM) crops: milestones and new advances in crop improvement. Theor. Appl. Genet. 129, 1639–1655. 10.1007/s00122-016-2747-6 27381849

[B63] Kavuličová J. Kaduková J. Ivánová D. (2012). The evaluation of heavy metal toxicity in plants using the biochemical Tests. 11, 101–110. 10.2478/v10296-012-0011-2

[B64] Kawashima C. G. Noji M. Nakamura M. Ogra Y. Suzuki K. T. Saito K. (2004). Heavy metal tolerance of transgenic tobacco plants over-expressing cysteine synthase. Biotechnol. Lett. 26, 153–157. 10.1023/B:BILE.0000012895.60773.ff 15000484

[B65] Khalid S. Shahid M. Niazi N. K. Murtaza B. Bibi I. Dumat C. (2017). A comparison of technologies for remediation of heavy metal contaminated soils. J. Geochem. Explor. 182, 247–268. 10.1016/j.gexplo.2016.11.021

[B66] Kidd P. Mench M. Alvarez-Lopez V. Bert V. Dimitriou I. Friesl-Hanl W.. (2015). Agronomic practices for improving gentle remediation of trace element-contaminated soils. Int. J. Phytoremediation 17, 1005–1037. 10.1080/15226514.2014.1003788 25581041

[B67] Kille P. Winge D. R. Harwood J. L. Kay J. (1991). A plants metallothionein produced in *Escherichia coli*. FEBS Lett. 295, 171–175. 10.1016/0014-5793(91)81411-Z 1765150

[B68] Kim Y. N. Kim J. S. Seo S. G. Lee Y. Baek S. W. Kim I. S.. (2011). Cadmium resistance in tobacco plants expressing the MuSI gene. Plant Biotechnol. Rep. 5, 323–329. 10.1007/s11816-011-0186-z 22031812 PMC3191292

[B69] Kiyono M. Oka Y. Sone Y. Tanaka M. Nakamura R. Sato M. H.. (2012). Expression of the bacterial heavy metal transporter MerC fused with a plant SNARE, SYP121, in *Arabidopsis thaliana* increases cadmium accumulation and tolerance. Planta 235, 841–850. 10.1007/s00425-011-1543-4 22089884

[B70] Koprivova A. Kopriva S. Jager D. Will B. Jouanin L. Rennenberg H. (2002). Evaluation of transgenic poplars over-expressing enzymes of glutathione synthesis for phytoremediation of cadmium. Plant Biol. 4, 664–670. 10.1055/s-2002-37399

[B71] Kotrba P. Najmanova J. Macek T. Ruml T. Mackova M. (2009). Genetically modified plants in phytoremediation of heavy metal and metalloid soil and sediment pollution. Biotechnol. Adv. 27, 799–810. 10.1016/j.biotechadv.2009.06.003 19567265

[B72] Krystofova O. Zitka O. Krizkova S. Hynek D. Shestivska V. Adam V. (2012). Accumulation of cadmium by transgenic tobacco plants (*Nicotiana tabacum L*.) carrying yeast metallothionein gene revealed by electrochemistry. Int. J. Electrochem. Sci. 7, 886–907.

[B73] Krzciuk K. Gałuszka A. (2015). Prospecting for hyperaccumulators of trace elements: a review. Crit. Rev. Biotechnol. 35, 522–532. 10.3109/07388551.2014.922525 24938121

[B74] Krzesłowska M. (2011). The cell wall in plant cell response to trace metals: polysaccharide remodeling and its role in defense strategy. Acta Physiol. Plant. 33, 35–51. 10.1007/s11738-010-0581-z

[B75] Lang M. Hao M. Fan Q. Wang W. Mo S. Zhao W. C.. (2011). Functional characterization of BjCET3 and BjCET4, two new cation-efflux transporters from *Brassica juncea L*. J. Exp. Bot. 62, 4467–4480. 10.1093/jxb/err137 21652531 PMC3170545

[B76] Le Gall H. Philippe F. Domon J.-M. Gillet F. Pelloux J. Rayon C. (2015). Cell wall metabolism in response to abiotic stress. Plants 4:112. 10.3390/plants4010112 27135320 PMC4844334

[B77] Lee J. Bae H. Jeong J. Lee J. Y. Yang Y. Y. Hwang I. (2003). Functional expression of a bacteral heavy metal transporter in arabidopsis enhances resistance to and decrease uptake of heavy metals. Plant Physiol. 133, 589–596. 10.1104/pp.103.021972 14512517 PMC219035

[B78] Leitenmaier B. Küpper H. (2013). Compartmentation and complexation of metals in hyperaccumulator plants. Front. Plant Sci. 4:374. 10.3389/fpls.2013.00374 24065978 PMC3778397

[B79] Leszczyszyn O. I. Imam H. T. Blindauer C. A. (2013). Diversity and distribution of plant metallothioneins: a review of structure, properties and functions. Metallomics 5, 1146–1169. 10.1039/c3mt00072a 23694960

[B80] Li J. C. Guo J. B. Xu W. Z. Ma M. (2006a). Enhanced cadmium accumulation in transgenic tobacco expressing the phytochelatin synthase gene of *Cynodon dactylon L. J. Integr*. Plant Biol. 48, 928–937. 10.1111/j.1744-7909.2006.00314.x

[B81] Li Y. J. Heaton A. C. P. Carreira L. Meagher R. B. (2006b). Enhanced tolerance to and accumulation of mercury, but not arsenic, in plants overexpressing two enzymes required for thiol peptide synthesis. Physiol. Plant. 128, 48–57. 10.1111/j.1399-3054.2006.00732.x

[B82] Li Z. S. Szczypka M. Lu Y. P. Thiele D. J. Rea P. A. (1996). The yeast cadmium factor protein (YCF1) is a vacuolar glutathione S-conjugate pump. J. Biol. Chem. 271, 6509–6517. 10.1074/jbc.271.11.6509 8626454

[B83] Liang Zhu Y. Pilon-Smits, E. A. H. Jouanin L. Terry N. (1999). Overexpression of glutathione synthetase in Indian mustard enhances cadmium accumulation and tolerance. Plant Physiol. 119, 73–79. 9880348 10.1104/pp.119.1.73 PMC32244

[B84] Liu D. An Z. Mao Z. Ma L. Lu Z. (2015). Enhanced heavy metal tolerance and accumulation by transgenic sugar beets expressing *Streptococcus thermophilus* StGCS-GS in the presence of Cd, Zn and Cu alone or in combination. PLoS ONE 10:e0128824 10.1371/journal.pone.0128824 26057126 PMC4461316

[B85] Liu X. Wu S. Xu J. Sui C. Wei J. (2017). Application of CRISPR/Cas9 in plant biology. Acta Pharm. Sin. B 7, 292–302. 10.1016/j.apsb.2017.01.002 28589077 PMC5443236

[B86] Liu Z. Gu C. Chen F. Yang D. Wu K. Chen S.. (2012). Heterologous expression of a *Nelumbo nucifera* phytochelatin synthase gene enhances cadmium tolerance in *Arabidopsis thaliana*. Appl. Biochem. Biotechnol. 166, 722–734. 10.1007/s12010-011-9461-2 22161260

[B87] Macci C. Doni S. Peruzzi E. Bardella S. Filippis G. Ceccanti B.. (2013). A real-scale soil phytoremediation. Biodegradation 24, 521–538. 10.1007/s10532-012-9608-z 23179352

[B88] Macci C. Peruzzi E. Doni S. Poggio G. Masciandaro G. (2016). The phytoremediation of an organic and inorganic polluted soil: a real scale experience. Int. J. Phytoremediation 18, 378–386. 10.1080/15226514.2015.1109595 26555402

[B89] Macek T. Kotrba P. Svatos A. Novakova M. Demnerova K. Mackova M. (2008). Novel roles for genetically modified plants in environmental protection. Trends Biotechnol. 26, 146–152. 10.1016/j.tibtech.2007.11.009 18243383

[B90] Macek T. Macková M. Kás J. (2000). Exploitation of plants for the removal of organics in environmental remediation. Biotechnol. Adv. 18, 23–34. 10.1016/S0734-9750(99)00034-8 14538117

[B91] Macek T. Novakova M. Kotrba P. Viktorova J. Lovecka P. Fiser J. (2013). Genetically Modified Plants Designed for Phytoremediation of Toxic Organic And Inorganic Contaminants. Boca Raton, FL: Crc Press-Taylor & Francis Group.

[B92] Macek T. Uhlik O. Jecna K. Novakova M. Lovecka P. Rezek J. (2009). Advances in phytoremediation and rhizoremediation, in Soil Biology, 17 Edn, ed Varma A. (Berlin: Springer), 257–277.

[B93] Mackova M. Barriault D. Francova K. Sylvestre M. Moder M. Vrchotova B. (2006a). Phytoremediation of polychlorinated biphenyls, in Focus on Biotechnology, 9A Edn., eds Mackova M. Dowling D. Macek T. (Dordrecht: Springer), 143–167.

[B94] Mackova M. Dowling D. Macek T. (2006b). Phytoremediation and Rhizoremediation. Theoretical Background. Dordrecht: Springer.

[B95] Marmiroli N. Marmiroli M. Maestri E. (2006). Phytoremediation and phytotechnologies: a review for the present and the future Soil Water Pollmoniter. Protec. Remed. 69, 403–416. 10.1007/978-1-4020-4728-2_26

[B96] Martínez M. Bernal P. Almela C. Vélez D. García-Agustín P. Serrano R.. (2006). An engineered plant that accumulates higher levels of heavy metals than *Thlaspi caerulescens*, with yields of 100 times more biomass in mine soils. Chemosphere 64, 478–485. 10.1016/j.chemosphere.2005.10.044 16337669

[B97] McGrath S. P. Zhao F. J. (2003). Phytoextraction of metals and metalloids from contaminated soils. Curr. Opin. Biotechnol. 14, 277–282. 10.1016/S0958-1669(03)00060-0 12849780

[B98] McGrath S. P. Zhao F. J. Lombi E. (2002). Phytoremediation of metals, metalloids, and radionuclides, in Advances in Agronomy, ed Sparks D. L. (San Diego, CA; London: Academic Press Inc.,; Academic Press Ltd.), 92101–4495.

[B99] Meister A. (1988). Glutathione metabolism and its selective modification. J. Biol. Chem. 263, 17205–17208. 3053703

[B100] Mench M. Schwitzguébel J. P. Schroeder P. Bert V. Gawronski S. Gupta S. (2009). Assessment of successful experiments and limitations of phytotechnologies: contaminant uptake, detoxification and sequestration, and consequences for food safety. Environ. Sci. Pollut. Res. 16, 876–900. 10.1007/s11356-009-0252-z 19823886

[B101] Milner M. J. Kochian L. V. (2008). Investigating heavy-metal hyperaccumulation using *Thlaspi caerulescens* as a model system. Ann. Bot. 102, 3–13. 10.1093/aob/mcn063 18440996 PMC2712422

[B102] Mosa K. A. Saadoun I. Kumar K. Helmy M. Dhankher O. P. (2016). Potential biotechnological strategies for the cleanup of heavy metals and metalloids. Front. Plant Sci. 7:303. 10.3389/fpls.2016.00303 27014323 PMC4791364

[B103] Nishiyama Y. Yanai J. Kosaki T. (2005). Potential of *Thlaspi caerulescens* for cadmium phytoremediation: comparison of two representative soil types in Japan under different planting frequencies. Soil Sci. Plant Nutr. 51, 827–834. 10.1111/j.1747-0765.2005.tb00117.x

[B104] Nixon D. J. Stephens W. Tyrrel S. F. Brierley E. D. R. (2001). The potential for short rotation energy forestry on restored landfill caps. Bioresour. Technol. 77, 237–245. 10.1016/S0960-8524(00)00081-X 11272010

[B105] Noctor G. Strohm M. Jouanin L. Kunert K. J. Foyer C. H. Rennenberg H. (1996). Synthesis of glutathione in leaves of transgenic poplar overexpressing γ-glutamylcysteine synthetase. Plant Physiol. 112, 1071–1078. 10.1104/pp.112.3.1071 12226433 PMC158033

[B106] Palmgren M. G. Clemens S. Williams L. E. Kraemer U. Borg S. Schjorring J. K.. (2008). Zinc biofortification of cereals: problems and solutions. Trends Plant Sci. 13, 464–473. 10.1016/j.tplants.2008.06.005 18701340

[B107] Pavlikova D. Macek T. Mackova M. Sura M. Szakova J. Tlustos P. (2004). The evaluation of cadmium, zinc and nickel accumulation ability of transgenic tobacco bearing different transgenes. Plant Soil Environ. 50, 513–517. 10.17221/4067-PSE

[B108] Peuke A. D. Rennenberg H. (2005). Phytoremediation-Molecular biology, requirements for application, environmental protection, public attention and feasibility. EMBO Rep. 6, 497–501. 10.1038/sj.embor.7400445 15940279 PMC1369103

[B109] Pianelli K. Mari S. Marques L. Lebrun M. Czernic P. (2005). Nicotianamine over-accumulation confers resistance to nickel in *Arabidopsis thaliana*. Transg. Res. 14, 739–748. 10.1007/s11248-005-7159-3 16245165

[B110] Pilon-Smits E. A. H. Hwang S. B. Lytle C. M. Zhu Y. L. Tai J. C. Bravo R. C.. (1999). Overexpression of ATP sulfurylase in Indian mustard leads to increased selenate uptake, reduction, and tolerance. Plant Physiol. 119, 123–132. 10.1104/pp.119.1.123 9880353 PMC32211

[B111] Pilon-Smits E. A. H. Leduc D. L. (2009). Phytoremediation of selenium using transgenic plants. Curr. Opin. Biotechnol. 20, 207–212. 10.1016/j.copbio.2009.02.001 19269806

[B112] Postrigan B. N. Knyazev A. B. Kuluev B. R. Yakhin O. I. Chemeris A. V. (2012). Expression of the synthetic phytochelatin gene in tobacco. Russ. J. Plant Physiol. 59, 275–280. 10.1134/S1021443712020136

[B113] Prasad M. N. V. Freitas H. M. D. (2003). Metal hyperaccumulation in plants-biodiversity prospecting for phytoremediation technology. Electron. J. Biotechnol. 6, 285–321. 10.2225/vol6-issue3-fulltext-6

[B114] Rascio N. Navari-Izzo F. (2011). Heavy metal hyperaccumulating plants: how and why do they do it? and what makes them so interesting? Plant Sci. 180, 169–181. 10.1016/j.plantsci.2010.08.016 21421358

[B115] Raskin I. Smith R. D. Salt D. E. (1997). Phytoremediation of metals: using plants to remove pollutants from the environment. Curr. Opin. Biotechnol. 8, 221–226. 10.1016/S0958-1669(97)80106-1 9079727

[B116] Reeves R. (2006). Hyperaccumulation of trace elements by plants, in Phytoremediation of Metal-Contaminated Soils, eds Morel J. L. Echevarria G. Goncharova N. (Dordrecht: Springer), 25–52.

[B117] Reeves R. D. Schwartz C. Morel J. L. Edmondson J. (2001). Distribution and metal-accumulating behavior of *Thlaspi caerulescens* and associated metallophytes in France. Int. J. Phytoremediation 3, 145–172. 10.1080/15226510108500054

[B118] Rizwan M. Ali S. Qayyum M. F. Ok Y. S. Zia-ur-Rehman M. Abbas Z.. (2017). Use of Maize (*Zea mays L.)* for phytomanagement of Cd-contaminated soils: a critical review. Environ. Geochem. Health 39, 259–277. 10.1007/s10653-016-9826-0 27061410

[B119] Robinson B. H. Chiarucci A. Brooks R. R. Petit D. Kirkman J. H. Gregg P. E. H. (1997). The nickel hyperaccumulator plant *Alyssum bertolonii* as a potential agent for phytoremediation and phytomining of nickel. J. Geochem. Explor. 59, 75–86. 10.1016/S0375-6742(97)00010-1

[B120] Robinson B. H. Leblanc M. Petit D. Brooks R. R. Kirkman J. H. Gregg P. E. H. (1998). The potential of *Thlaspi caerulescens* for phytoremediation of contaminated soils. Plant Soil 203, 47–56. 10.1023/A:1004328816645

[B121] Rodriguez H. Vessely S. Shah S. Glick B. R. (2008). Effect of a nickel-tolerant ACC deaminase-producing *Pseudomonas* strain on growth of nontransformed and transgenic canola plants. Curr. Microbiol. 57, 170–174. 10.1007/s00284-008-9181-1 18560939

[B122] Rudolph A. Becker R. Scholz G. Procházka Ž. Toman J. Macek T. (1985). The occurrence of the amino acid nicotianamine in plants and microorganisms. A reinvestigation. Biochemie Physiologie der Pflanzen 180, 557–563. 10.1016/S0015-3796(85)80036-6

[B123] Rugh C. L. Wilde H. D. Stack N. M. Thompson D. M. Summers A. O. Meagher R. B. (1996). Mercuric ion reduction and resistance in transgenic *Arabidopsis thaliana* plants expressing a modified bacterial merA gene. Proc. Natl. Acad. Sci. U.S.A. 93, 3182–3187. 10.1073/pnas.93.8.3182 8622910 PMC39579

[B124] Sahi S. V. Bryant N. L. Sharma N. C. Singh S. R. (2002). Characterization of a lead hyperaccumulator shrub, *Sesbania drummondii*. Environ. Sci. Technol. 36, 4676–4680. 10.1021/es020675x 12433181

[B125] Salt D. E. Blaylock M. Kumar N. Dushenkov V. Ensley B. D. Chet I.. (1995). Phytoremediation-a novel strategy for the removal of toxic metals from the environment using plants. Biotechnology 13, 468–474. 9634787 10.1038/nbt0595-468

[B126] Salt D. E. Smith R. D. Raskin I. (1998). Phytoremediation. Annu. Rev. Plant Physiol. Plant Mol. Biol. 49, 643–668. 10.1146/annurev.arplant.49.1.643 15012249

[B127] Sandermann H. (1994). Higher-plant metabolism of xenobiotics-the green liver concept. Pharmacogenetics 4, 225–241. 10.1097/00008571-199410000-00001 7894495

[B128] Sanvido O. Romeis J. Gathmann A. Gielkens M. Raybould A. Bigler F. (2012). Evaluating environmental risks of genetically modified crops: ecological harm criteria for regulatory decision-making. Environ. Sci. Policy 15, 82–91. 10.1016/j.envsci.2011.08.006

[B129] Sarma H. (2011). Metal hyperaccumulation in plants: a review focusing on phytoremediation technology. J. Environ. Sci. Technol. 4, 118–138. 10.3923/jest.2011.118.138

[B130] Sasaki Y. Hayakawa T. Inoue C. Miyazaki A. Silver S. Kusano T. (2006). Generation of mercury-hyperaccumulating plants through transgenic expression of the bacterial mercury membrane transport protein MerC. Transgenic Res. 15, 615–625. 10.1007/s11248-006-9008-4 16830224

[B131] Schnoor J. L. Light L. A. McCutcheon S. C. Wolfe N. L. Carreia L. H. (1995). Phytoremediation of organic and nutrient contaminants. Environ. Sci. Technol. 29, 318A−323A. 10.1021/es00007a747 22667744

[B132] Sheoran V. Sheoran A. S. Poonia P. (2009). Phytomining: a review. Miner. Eng. 22, 1007–1019. 10.1016/j.mineng.2009.04.001

[B133] Shim D. Kim S. Choi Y.-I. Song W.-Y. Park J. Youk E. S.. (2013). Transgenic poplar trees expressing yeast cadmium factor 1 exhibit the characteristics necessary for the phytoremediation of mine tailing soil. Chemosphere 90, 1478–1486. 10.1016/j.chemosphere.2012.09.044 23062827

[B134] Shukla D. Kesari R. Mishra S. Dwivedi S. Tripathi R. D. Nath P. (2012). Expression of phytochelatin synthase from aquatic macrophyte *Ceratophyllum demersum L*. enhances cadmium and arsenic accumulation in tobacco. Plant Cell Rep. 31, 1687–1699. 10.1007/s00299-012-1283-3 22614255

[B135] Silver S. Phung le T. (2005). A bacterial view of the periodic table: genes and proteins for toxic inorganic ions. J. Indust. Microbiol. Biotechnol. 32, 587–605. 10.1007/s10295-005-0019-6 16133099

[B136] Silver S. Phung L. T. (2005). Genes and enzymes involved in bacterial oxidation and reduction of inorganic arsenic. Appl. Environ. Microbiol. 71, 599–608. 10.1128/AEM.71.2.599-608.2005 15691908 PMC546828

[B137] Singh S. Parihar P. Singh R. Singh V. P. Prasad S. M. (2016). Heavy metal tolerance in plants: role of transcriptomics, proteomics, metabolomics, and ionomics. Front. Plant Sci. 6:1143. 10.3389/fpls.2015.01143 26904030 PMC4744854

[B138] Singh S. P. Keller B. Gruissem W. Bhullar N. K. (2017). Rice NICOTIANAMINE SYNTHASE 2 expression improves dietary iron and zinc levels in wheat. Theor. Appl. Genet. 130, 283–292. 10.1007/s00122-016-2808-x 27722771 PMC5263203

[B139] Sorrentino M. C. Capozzi F. Amitrano C. Giordano S. Arena C. Spagnuolo V. (2018). Performance of three cardoon cultivars in an industrial heavy metal-contaminated soil: effects on morphology, cytology and photosynthesis. J. Hazard. Mater. 351, 131–137. 10.1016/j.jhazmat.2018.02.044 29529561

[B140] Suman J. Kotrba P. Macek T. (2014). Putative P-1B-type ATPase from the bacterium Achromobacter xylosoxidans A8 alters Pb2+/Zn2+/Cd2+-resistance and accumulation in Saccharomyces cerevisiae. Biochim. Biophys. Acta-Biomemb. 1838, 1338–1343. 10.1016/j.bbamem.2014.01.023 24491492

[B141] Suryawanshi V. Talke I. N. Weber M. Eils R. Brors B. Clemens S.. (2016). Between-species differences in gene copy number are enriched among functions critical for adaptive evolution in *Arabidopsis halleri*. BMC Genomics 17:1034. 10.1186/s12864-016-3319-5 28155655 PMC5259951

[B142] Tangahu B. V. Sheikh Abdullah S. R. Basri H. Idris M. Anuar N. Mukhlisin M. (2011). A review on heavy metals (As, Pb, and Hg) uptake by plants through phytoremediation. Int. J. Chem. Eng. 2011:939161 10.1155/2011/939161

[B143] Thomas J. C. Davies E. C. Malick F. K. Endreszl C. Williams C. R. Abbas M.. (2003). Yeast metallothionein in transgenic tobacco promotes copper uptake from contaminated soils. Biotechnol. Prog. 19, 273–280. 10.1021/bp025623q 12675559

[B144] Tommey A. M. Shi J. Lindsay W. P. Urwin P. E. Robinson N. J. (1991). Expression of the pea gene PSMTA in *E. coli*. Metal-binding properties of the expressed protein. FEBS Lett. 292, 48–52. 1959626 10.1016/0014-5793(91)80831-m

[B145] Trijatmiko K. R. Dueñas C. Tsakirpaloglou N. Torrizo L. Arines F. M. Adeva C.. (2016). Biofortified indica rice attains iron and zinc nutrition dietary targets in the field. Sci. Rep. 6:19792. 10.1038/srep19792 26806528 PMC4726380

[B146] Tsatsakis A. M. Nawaz M. A. Kouretas D. Balias G. Savolainen K. Tutelyan V. A.. (2017). Environmental impacts of genetically modified plants: a review. Environ. Res. 156, 818–833. 10.1016/j.envres.2017.03.011 28347490

[B147] Unterbrunner R. Puschenreiter M. Sommer P. Wieshammer G. Tlustoš P. Zupan M.. (2007). Heavy metal accumulation in trees growing on contaminated sites in Central Europe. Environ. Pollut. 148, 107–114. 10.1016/j.envpol.2006.10.035 17224228

[B148] van der Ent A. Baker A. J. M. Reeves R. D. Pollard A. J. Schat H. (2013). Hyperaccumulators of metal and metalloid trace elements: facts and fiction. Plant Soil 362, 319–334. 10.1007/s11104-012-1287-3

[B149] Van Slycken S. Witters N. Meers E. Peene A. Michels E. Adriaensen K.. (2013a). Safe use of metal-contaminated agricultural land by cultivation of energy maize (*Zea mays*). Environ. Pollut. 178, 375–380. 10.1016/j.envpol.2013.03.032 23607942

[B150] Van Slycken S. Witters N. Meiresonne L. Meers E. Ruttens A. Van Peteghem P.. (2013b). Field evaluation of willow under short rotation coppice for phytomanagement of metal-polluted agricultural soils. Int. J. Phytoremediation 15, 677–689. 10.1080/15226514.2012.723070 23819267

[B151] Vangronsveld J. Herzig R. Weyens N. Boulet J. Adriaensen K. Ruttens A.. (2009). Phytoremediation of contaminated soils and groundwater: lessons from the field. Environ. Sci. Pollut. Res. 16, 765–794. 10.1007/s11356-009-0213-6 19557448

[B152] Verbruggen N. Hermans C. Schat H. (2009). Molecular mechanisms of metal hyperaccumulation in plants. N. Phytol. 181, 759–776. 10.1111/j.1469-8137.2008.02748.x 19192189

[B153] Volk T. A. Verwijst T. Tharakan P. J. Abrahamson L. P. White E. H. (2004). Growing fuel: a sustainability assessment of willow biomass crops. Front. Ecol. Environ. 2, 411–418. 10.1890/1540-9295(2004)002[0411:GFASAO]2.0.CO;2

[B154] Vyslouzilova M. Puschenreiter M. Wieshammer G. Wenzel W. W. (2006). Rhizosphere characteristics, heavy metal accumulation and growth performance of two willow (*Salix x rubens*) clones. Plant Soil Environ. 52, 353–361. 10.17221/3452-PSE

[B155] Wang L. Ji B. Hu Y. Liu R. Sun W. (2017). A review on in situ phytoremediation of mine tailings. Chemosphere 184, 594–600. 10.1016/j.chemosphere.2017.06.025 28623832

[B156] Wojas S. Hennig J. Plaza S. Geisler M. Siemianowski O. Sklodowska A.. (2009). Ectopic expression of *Arabidopsis* ABC transporter MRP7 modifies cadmium root-to-shoot transport and accumulation. Environ. Pollut. 157, 2781–2789. 10.1016/j.envpol.2009.04.024 19467746

[B157] Wu Q. Shigaki T. Williams K. A. Han J. S. Kim C. K. Hirschi K. D.. (2011). Expression of an Arabidopsis Ca2+/H+ antiporter CAX1 variant in petunia enhances cadmium tolerance and accumulation. J. Plant Physiol. 168, 167–173. 10.1016/j.jplph.2010.06.005 20633955

[B158] Wu T.-Y. Gruissem W. Bhullar N. K. (2018). Facilitated citrate-dependent iron translocation increases rice endosperm iron and zinc concentrations. Plant Sci. 270, 13–22. 10.1016/j.plantsci.2018.02.002 29576065

[B159] Xiao S. Gao W. Chen Q. F. Ramalingam S. Chye M. L. (2008). Overexpression of membrane-associated acyl-CoA-binding protein ACBP1 enhances lead tolerance in *Arabidopsis*. Plant J. 54, 141–151. 10.1111/j.1365-313X.2008.03402.x 18182029

[B160] Xu W. Lu G. Dang Z. Liao C. Chen Q. Yi X. (2013). Uptake and distribution of Cd in sweet maize grown on contaminated soils: a field-scale study. Bioinorgan. Chem. Appl. 2013:959764 10.1155/2013/959764 PMC3856120 24348276

[B161] Yankov B. Delibaltova V. Bojinov M. (2000). Contents of Cu, Zn, Cd and Pb in the vegetative organs of cotton cultivars from industrially polluted region. Rasteniev"dni Nauki 37, 525–531.

[B162] Zhang H. Y. Xu W. Z. Dai W. T. He Z. Y. Ma M. (2006). Functional characterization of cadmium-responsive garlic gene AsMT2b: a new member of metallothionein family. Chinese Sci. Bull. 51, 409–416. 10.1007/s11434-006-0409-9

[B163] Zhang Y. Zhao L. H. Wang Y. Yang B. Y. Chen S. Y. (2008). Enhancement of heavy metal accumulation by tissue specific co-expression of iaaM and ACC deaminase genes in plants. Chemosphere 72, 564–571. 10.1016/j.chemosphere.2008.03.043 18471863

[B164] Zhao C. Qiao M. Yu Y. Xia G. Xiang F. (2010). The effect of the heterologous expression of Phragmites australis gamma-glutamylcysteine synthetase on the Cd2+ accumulation of *Agrostis palustris*. Plant Cell Environ. 33, 877–887. 10.1111/j.1365-3040.2009.02113.x 20051038

[B165] Zheljazkov V. D. Nielsen N. E. (1996a). Effect of heavy metals on peppermint and cornmint. Plant Soil 178, 59–66. 10.1007/BF00011163

[B166] Zheljazkov V. D. Nielsen N. E. (1996b). Studies on the effect of heavy metals (Cd, Pb, Cu, Mn, Zn and Fe) upon the growth, productivity and quality of lavender (*Lavandula angustifolia* Mill.) production. J. Essent. Oil Res. 8, 259–274.

[B167] Zhu Y. L. Pilon-Smits E. A. H. Tarun A. S. Weber S. U. Jouanin L. Terry N. (1999). Cadmium tolerance and accumulation in Indian mustard is enhanced by overexpressing γ-glutamylcysteine synthetase. Plant Physiol. 121, 1169–1177. 10594104 10.1104/pp.121.4.1169 PMC59484

